# On the Treatment of Some Forms of Menorrhagia

**Published:** 1872-06

**Authors:** Lombe Atthill

**Affiliations:** University of Dublin; Fellow and Examiner in Midwifery, King and Queen’s College of Physicians, Obstetric Physician to the Adelaide Hospital, Dublin, &c.


### ON THE TREATMENT OF SOME FORMS OF MENORRHAGIA.

BY LOMBE ATTHILL, M. D., UNIVERSITY OF DUBLIN;
Fellow and Examiner in Midwifery, King and Queen's College of Physicians,
Obstetric Physician to the Adelaide Hospital, Dublin, &c.

In this paper Dr. Atthill confines himself to the consideration of the treatment most suitable to cases of menorrhagia when occurring in connection with, or dependent on, sub-involution of the uterus, or granular ulceration of the cervix, or an unhealthy condition of the mucous membrane lining the body of the uterus.

Defective involution of the uterus after labor or abortion occupies a prominent place among the causes giving rise to excessive menstruation. That this should be the case is but natural, for not only is there, when sub-involution exists, an undue amount of blood present in the organ, but also the relaxed condition of the uterine tissue favors its exudation, and therefore, when the periodic determination of blood to the uterus takes place, as it occurs at each menstrual period, the moderate flow which should relieve that congestion, becomes a profuse discharge, and often an exhausting drain. But the mischief resulting from sub-involution does not end here, for that abnormal state of the uterus predisposes to that unhealthy condition known as "granular ulceration" of the os and cervix uteri, a condition in which the mucous membrane of the canal of the cervix is hypertrophied, becomes exceedingly vascular, and is often everted to a considerable extent, a condition which increases the previously existing tendency to hemorrhage. Thus in not a few cases do we find that two causes present in the same patient. The author narrates a case which affords a well-marked instance of this, in which sub-involution was manifestly the primary cause of the menorrhagia, the ulceration being altogether secondary. In many cases sub-involution exists alone, or, on the other hand, ulceration may exist alone, either condition being fully sufficient to give origin to severe menorrhagia.

As an instance of the former the following serves as an example:

F. L., aged twenty-four, married about a year, was a delicate young woman of lymphatic temperament. Menstruation had always been profuse, especially if she took walking exercise, or exerted herself during the flow. She became pregnant after the occurrence of the second menstrual period following her marriage, but having imprudently taken a long and fatiguing walk, she aborted at about the eighth week; the two subsequent menstrual periods were so profuse as to reduce her to a state of extreme debility. Ergot, gallic acid, etc., failed to do good. On examining her after the termination of these periods, the uterus proved to be considerably elongated, the sound passing to the depth of three inches and a half; there did not exist any ulceration. The history of the case being altogether against the supposition of the existence of polypus, Dr. Atthill came to the conclusion that the menorrhagia depended on sub-involution; in fact, that the uterus had never regained its normal size and tone since the miscarriage, which had occurred two months previously. He, therefore, decided on carrying out a plan of treatment, the value of which he has repeatedly tested—viz: the introduction up to the fundus of the uterus of ten grains of the solid nitrate of silver, and leaving it to dissolve there. This was accordingly done. The application produced considerable pain, which lasted five or six hours, but no further unpleasant results followed. Dr. Atthill confined this patient to bed for several days, but then allowed her to go about. Menstruation appeared at the regular time, and was moderate in quantity; and she became pregnant immediately afterwards.

Dr. Atthill calls attention especially to this case; first, as illustrating the occurrence of sub-involution as a result of abortion—a fact which is overlooked by many; next, as showing the dangerous menorrhagia which may depend on this condition of the uterus; and, thirdly, as proving the excellent results which follow the treatment adopted. Ergot, gallic acid, and indeed, all other medicines, will frequently fail to check menorrhagia depending on sub-involution; and we must have recourse to treatment directed to the uterus itself; we must stimulate the organ to set up that healthy action by which it regains its normal size after pregnancy has terminated—a process to which Sir J. Simpson, has applied the term "involution." With this view, the author unhesitatingly advocates the adoption of the treatment practiced in the preceding case. He knows no other so efficacious.

The mode of carrying it out is simple. The instrument known as Sir James Simpson's "*uterine porte caustique*" is introduced into the uterus just as an ordinary uterine sound. This little instrument consists of a hollow silver tube, in size and shape closely resembling a sound; it contains a flexible stilette, which it fits accurately. As soon as its point is found to have reached the fundus of the uterus, the stilette is withdrawn, and through the instrument is pushed up, by means of the stilette, a piece of solid nitrate of silver, reduced to the requisite size and weight, till it is fairly lodged in the cavity of the uterus. In doing this, there is but one caution requisite to be attended to: namely, that as soon as the piece of nitrate of silver has reached the extremity of the *porte caustique*, and before it is finally pushed out of the instrument—a point of which we can always be certain by observing how much of the stilette remains still unintroduced—the instrument should be withdrawn to the extent of about half an inch; for if this precaution be not observed, it is possible that the nitrate of silver might be forced into the substance of the uterine wall, instead of being left free in its cavity—an accident which though possible, is very unlikely to occur.

Menorrhagia resulting from ulceration of the os and cervix uteri is also of frequent occurrence. Mere abrasion of the lips of uteri is not sufficient to produce menorrhagia; but that unhealthy, spongy condition of the os and cervix, in which the mucous membrane lining its canal becoming hypertrophied and thickened, bleeds on the slightest touch, the os being patulous and the lips everted, is quite capable of originating severe menorrhagia.

"Mrs. B—, a young married woman, aged twenty-four, who had never been pregnant, stated that she had become greatly debilitated by the excessive loss which occurred at each menstrual period. Ergot and astringents were exhibited by the mouth, and astringent lotions injected into the vagina, without producing the least effect. The use of the speculum proved the existence of extensive granular ulceration of the os and cervix uteri. Now, in severe cases, such as the one I am referring to, the unhealthy condition of the mucous membrane extends at least as high as the os internum, and we will fail to effect a cure unless our treatment reach every portion of the diseased tissue; therefore, with the view of permitting the necessary application to be made to the whole extent of the cervical canal, I commenced my treatment by introducing two tents of compressed sea-tangle, two pieces being sufficient for the object I had in view, which was not to open the uterus to such an extent as to enable me to examine its cavity, but only to permit me to treat the entire of the cervical canal. I left these pieces *in situ* for twenty-four hours, and on withdrawing them after the lapse of that time, cauterized freely the whole diseased surface, with fuming nitric acid. This did not cause any pain. On examining the os uteri a few days subsequently, I found it in a much healthier state. *The menorrhagia was entirely checked and never returned;* and, although a considerable time elapsed before the uterus regained a healthy state, still the progress of the case was rapid and the cure perfect; the only treatment subsequently necessary being the occasional application of a twenty-grain solution of nitrate of silver to the os uteri, and at a later period, of a small blister to the sacrum; finally, not the slightest trace of the ulceration remained, and menstruation became in all respects normal."

The foregoing case illustrates perfectly the mode of treatment which Dr. Atthill, as a rule, adopts in cases of granular ulceration of the os and cervix uteri. Of course it is not always necessary to dilate the cervix uteri. If the case be recent, and we can satisfy ourselves that the unhealthy condition of the mucous membrane does not extend very high, the use of the solid nitrate of silver, of zinc points, or brushing the part over lightly with nitric acid, may be sufficient; but in the severe forms of the disease, such treatment will merely be palliative, and the only effectual one will be found to consist in that which the author advocates. If we meet with a case of menorrhagia in an otherwise healthy woman, which a careful vaginal examination proves not to depend on granular ulceration of the os and cervix or on sub-involution, it is our manifest duty to dilate the cervix and os internum, with the view of determining what the condition of the interior of the body of the organ may be. As a rule, the uterus is seldom, in these cases, much elongated, the increase being not more than to the extent of perhaps half an inch. This point is of importance in enabling us to decide as to the possible presence of an intra-uterine tumor; but the existence of these or of the condition under consideration, can only be solved by dilating the cervix and then passing the finger fairly up to the fundus of the uterus. It is surprising how little the patient suffers from this process, and how rapidly the os regains its natural size. No less remarkable is the entire absence of all unpleasant symptoms after a proceeding apparently so severe. Dr. Atthill has not the least hesitation in recommending the practice.

The method of dilating the cervix is now so well known that the author does not dwell on it. He has quite given up the use of sponge-tents, and invariably adopts the plan, recommended by Dr. Kidd, of introducing a number of pieces of sea-tangle bougies. These are much superior for the purposes to the sea-tangle tents; for this reason, that they can be cut to any desired length, and that length should be the depth of the uterus, as previously ascertained by the use of the sound. The number of pieces introduced must vary considerably. If the cervix be rigid, three or four will be as many as can be safely inserted; but if it be relaxed, double, or even treble, that number may be, with impunity, inserted. If the smaller number be used, they should be withdrawn after the lapse of a few hours and a larger number inserted; but, in any case, the os internum must be dilated to a size sufficient to admit of the passage of the top of the index finger. To effect this by no means easy matter, the first step, after having withdrawn the sea-tangle, is to seize the anterior lip with a vulsellum, and with it to draw the uterus well down. This should be done by an assistant; pressure should also at the same time be made on the fundus; by these means the uterus will have been brought so low that, unless the pelvis be very deep, the point of the finger will reach the very fundus. and we are enabled to discover the presence of even a very small polypus, should it exist, or to detect that so-called "granular" condition of the mucous membrane, a condition which communicates a rough, uneven feel to the finger. This condition of the mucous membrane, Dr. Atthill has several times met with. On the first occasion, he was disappointed, and indeed surprised, at failing to detect a polypus, which, as he had announced to the class, he expected to find. Since then, enlarged experience has taught him that this granular condition of the lining membrane of the body of the uterus is a by no means infrequent cause of menorrhagia, and he now looks on its detection as a possible result of the exploration of the cavity of the uterus. Dr. Atthill believes it to be the result of congestion or of sub-acute inflammation of the membrane lining the uterine cavity, which has resulted in producing a thickened, unhealthy, vascular condition of that membrane. To cure this condition, it is essential to destroy the so-called granulation, and to endeavor to excite a healthy action in the diseased part. With this view the author makes use of the strong nitric acid, applying it with great freedom over the entire of the inner surface of the uterus by means of a bit of lint fastened securely to a piece of wood or inserted through a loop of iron wire. The os should be brought into view by means of a duck-bill speculum, which also serves to protect the posterior wall of the vagina. While the anterior wall is guarded by the vulsellum, with which the anterior lip should be still held firmly, the stick or wire, armed with the lint saturated with the acid, should then be passed rapidly through the cervix, and swept freely but quickly round the interior of the uterus. Another piece of lint, soaked in water, should be then passed up to the os to protect the vagina from the irritation which any acrid discharge from the os uteri might cause; and the lip being freed from the vulsellum, and the speculum withdrawn, the patient may be left, the only after-treatment necessary being that the vagina should be syringed out daily with tepid water, and absolute rest in bed enjoined for some days. With due attention to these precautions, no ill effects need be dreaded.

Such is a brief outline of the treatment which Dr. Atthill adopts in cases of menorrhagia depending on the causes mentioned.—*British Medical Journal.*

---

### ON DYSMENORRHŒA.

BY LOMBE ATTHILL, M. D., DUBLIN,

Fellow and Examiner in Midwifery King and Queen's College of Physicians, Obstetric Physician to the Adelaide Hospital, Dublin.

